# TRC-Based High-Precision Spot Position Detection in Inter-Satellite Laser Communication

**DOI:** 10.3390/s20195649

**Published:** 2020-10-02

**Authors:** Qing Li, Hongyang Guo, Shaoxiong Xu, Yangjie Xu, Qiang Wang, Dong He, Zhenming Peng, Yongmei Huang

**Affiliations:** 1 Institute of Optics and Electronics, Chinese Academy of Sciences, No.1 Guangdian Road, Chengdu 610209, China; qiou9@163.com (Q.L.); guohongy93@163.com (H.G.); hitxsx@163.com (S.X.); xyj1289467369@163.com (Y.X.); qiangwang@ioe.ac.cn (Q.W.); hedong@ioe.ac.cn (D.H.); 2 School of Information and Communication Engineering, University of Electronic Science and Technology of China, No.2006 Xiyuan Ave, West Hi-Tech Zone, Chengdu 611731, China; pengzm_ioe@163.com; 3 Key Laboratory of Optical Engineering, Chinese Academy of Sciences, Chengdu 610209, China; 4 University of Chinese Academy of Sciences, Beijing 100049, China

**Keywords:** inter-satellite laser communication system, weak light-spot position detection, four-quadrant detector, time reversal convolution

## Abstract

Inter-satellite laser communication (Is-OWC) is one of the main space optical communication technologies currently studied in various countries. In recent years, a kind of Is-OWC communication terminal without independent beacon light has appeared. Such terminals do not have a separate beacon laser with a large divergence angle, but use a narrower communication beam to complete space capture and tracking. Therefore, the energy of the light beam divided by the acquisition, tracking, and aiming (ATP) system is greatly reduced. How to perform high-precision spot position detection under extremely low signal-to-noise ratio (SNR) is a problem that must be faced. Aiming to resolve this problem, this article proposes to use a cosine signal to modulate the intensity of the signal light, so as to convert the problem of detecting a weak light signal into the problem of detecting a line spectrum signal. The authors used the time reversal convolution (TRC) algorithm with a window function to suppress noise and enhance the spectrum line, so as to accurately detect the amplitudes of the weak photocurrents. Finally, by calculating the ratio of the photocurrent amplitude values, the precise spot position is obtained. In the experiment, when the output SNR of the four-quadrant detector (QD) is as low as −17.86 dB, the proposed method can still detect the spot position and the absolute error is limited within 0.0238 mrad.

## 1. Introduction

Inter-satellite laser communication (Is-OWC) is one of the main space optical communication technologies currently studied in many countries [1,2]. The satellite-based optical communication terminal detects the incident angle of the laser beam through the acquisition, tracking, and aiming (ATP) system and controls the optical antenna to aim at another communication terminal, thereby establishing a high-quality communication link. When faced with the requirements of low-orbit satellite networking, space optical communication terminals must be developed towards high communication rates and miniaturization [3]. In recent years, a kind of Is-OWC communication terminal without independent beacon light has appeared [4,5,6]. In such terminals, the ATP system splits part of the light beam from the signal beam as the beacon light to detect the angle of the incidence light. The signal beam energy obtained by the on-board terminal remains unchanged, but in order to ensure communication quality, the ATP system will not separate too much beam energy from the signal beam, so the energy of the beacon light entering the ATP system is greatly reduced [7,8,9]. How to perform high-precision spot position detection under extremely low signal-to-noise ratio (SNR) is a problem that must be faced.

To detect a weak light spot position, this article uses a four-quadrant detector (QD) which has a fast response speed and outputs four continuous photocurrents. However, QD is susceptible to thermal noise and background radiation noise. To suppress these two kinds of noise, Qian [10] and Narayanan [11] used pulse signals to modulate signal light at the transmitting end, and then used narrow-band filters and sampling pulses to select receive signals at the receiving end. However, when the center frequency of the narrowband filter and the frequency of the sampling pulse are the same, the SNR of the received signal is not improved. In the field of distance measurement, Makynen Anssi [12] used the modulated laser as the transmission signal, and performed the correlation operation between the modulation signal and the reflected signal. In the ATP system based on position sensitive detector (PSD), to suppress background light and dark current noise, Hu Zheng [13] used a square wave to modulate the beacon light, and used the same frequency square wave signal at the receiving end to perform correlation calculation with the photocurrent signal output by PSD, which improves the detection accuracy. However, this method requires high synchronization between the two signals. Yu Jiawei [14] used the Kalman filter to process the photocurrent signal output by the QD to improve the accuracy of spot position detection. Gao Siyuan [15] established the beam detection range model of the laser guidance system based on QD, calibrated the influence of temperature parameters on the beam detection range, and performed temperature compensation for this, which greatly improves the beam detection performance of QD. In the moving target tracking system based on QD, Zhang Wugang [16] used low-order least squares fitting and Kalman filter to improve the detection accuracy of the spot position under strong noise environment. However, the methods discussed in the literatures [14,16] cannot provide sufficient detection accuracy when the SNR is extremely low (such as −17 dB).

To solve this problem, this paper attempts to provide sufficient SNR gain from the perspective of line spectrum signal enhancement. Therefore, we proposed a new method based on the time reversal convolution (TRC) line spectrum enhancement algorithm. This method uses a cosine signal to modulate the intensity of light beam, and converts the photocurrent signal output by the QD into a line spectrum signal. Then, the TRC algorithm with window function is used to suppress the noise and provide higher SNR gain to accurately detect the amplitude of the photocurrent. In this way, the detection accuracy of the spot position can be improved. When the output SNR of the QD is as low as −17.86 dB, the proposed method can still detect the spot position and the absolute error is limited within 0.0238 mrad. 

## 2. The Performance of the No Independent Beacon Light ATP System

**Figure 1:**
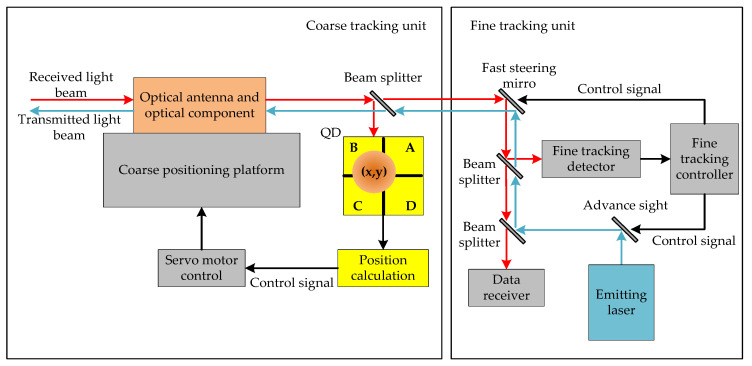
The no independent beacon light acquisition, tracking, and aiming (ATP) system.

As Figure 1 shows, the no independent beacon light Is-OWC terminal does not have a separate beacon laser with a large divergence angle, but uses a narrower signal light beam to complete space capture and tracking. Therefore, the energy of the light irradiating to the detector in the ATP system is extremely small. For example, when the light beam propagation distance is 50,000 km, the optical antenna aperture is 200 mm, and the beam divergence angle is 30 μrad, the geometric attenuation of the incident beam will reach 77 dB. In this case, the received SNR of the ATP system will be lower than −17 dB.

As a coarse tracking detector in the system, QD can be seen as the splicing of four independent avalanche photodiodes (APDs) in a rectangular coordinate system, with each APD corresponding to a coordinate quadrant. When incident light is irradiated on the photosensitive surface of QD, each APD will induce a corresponding photocurrent, and the amplitude of each photocurrent is proportional to the energy of the received light beam in each quadrant.

Since the proportional relationship of the output photocurrents can correspond to the coordinate position of the light spot, we can define a normalized variable that can quantitatively reflect the offset value of the light spot position relative to the coordinate origin, which is expressed as [17,18,19]:

(1) $$\Delta x=\frac{\left(I_{A}+I_{D})-(I_{B}+I_{C}\right)}{I_{A}+I_{B}+I_{C}+I_{D}}$$

(2) $$\Delta y=\frac{\left(I_{A}+I_{B})-(I_{C}+I_{D}\right)}{I_{A}+I_{B}+I_{C}+I_{D}}$$

where Δx and Δy are called the normalized offset value of the x-axis and y-axis, which quantitatively gives the offset position of the spot on the QD target surface relative to the center of the target surface.

It can be seen from Equations (1) and (2) that using QD to detect the spot position essentially calculates the distribution ratio of the light beam energy irradiated to the four APDs. Furthermore, literature [20] proposed that the spot position detection accuracy is affected by the factors such as spot position, spot radius and SNR. Therefore, it is necessary to find a way to effectively improve the output SNR of QD according to the characteristics of the noise distribution. 

In QD-based ATP systems, the main noise is background radiation noise and thermal noise. The both noises can be equivalent to Gaussian noise whose power spectrum density can be regarded as evenly distributed over the entire frequency band [21,22,23]. If a cosine signal is used to modulate the intensity of the transmitting light, the spectrum of the corresponding photocurrent signal output by the QD will be a line spectrum signal. Therefore, the energy of the photocurrent signal will be concentrated on a certain frequency point, thereby improving the SNR at the local frequency point, so as to improve the detection accuracy of the photocurrent amplitude.

## 3. The Response of QD to Modulated Light Beam 

A typical single frequency signal is a cosine (or a sine) signal, so the modulation signal can be defined as:

(3) $$d(t)=\cos(\Omega t+\varphi_{1})$$

The incident light field of the beacon light is *E_s_*, the light intensity *P_s_* can be written as:

(4) $$P_{s}(t)=E_{s}^{2}(t)=a^{2}\cdot \cos^{2}(wt+\varphi_{0})$$

where *a* is the light field intensity, *w* is the light frequency, and $\varphi_{0}$ is the phase of the incident light field. Then, the intensity of the modulated light can be expressed as:

(5) $$\begin{array}{ll} P_{s}(t) & =\frac{a^{2}}{2}[1+k_{p}\cdot d(t)]\cos^{2}(wt+\varphi_{0})\\ & =\frac{a^{2}}{2}[1+k_{p}\cdot \cos(\Omega t+\varphi_{1})]\cos^{2}(wt+\varphi_{0}) \end{array}$$

where *k_p_* is proportional coefficient. Then, the QD output current corresponding to the incident light signal is:

(6) $$I_{beam}=\eta \cdot \frac{a^{2}}{2}[1+k_{p}\cdot \cos(\Omega t+\varphi_{1})]$$

where η is the photoelectric conversion coefficient. It can be seen from Equation (6) that the output photocurrent contains the information of the modulation signal *d*(*t*). After passing through the AC coupling circuit, the photocurrent signal become a single frequency cosine signal, which can be rewritten as a voltage form:

(7) $$V(t)=s(t)+n(t)=KA\cos(\Omega t+\varphi_{1})+n(t)$$

where *K* is the total gain of the signal channel from the QD output to the analog-to-digital converter (ADC) and $A=k_{p}\cdot \eta \cdot a^{2}/2$ is the signal amplitude. *s*(*t*) responses to the light signal received by QD; *n*(*t*) is the noise. According to Euler’s formula, *s*(*t*) can be transformed as:

(8) $$\begin{array}{ll} s(t) & =\frac{KA}{2}(e^{j(\Omega +\phi_{1})}+e^{-j(\Omega +\phi_{1})})\\ & =\frac{KA}{2}(e^{j\phi_{1}}\cdot e^{j\Omega }+e^{-j\phi_{1}}\cdot e^{-j\Omega }) \end{array}$$

After Fourier transform, we can get:

(9) $$S(W)=\frac{KA}{2}[e^{j\phi_{1}}\cdot \delta (W+\Omega )+e^{-j\phi_{1}}\cdot \delta (W-\Omega )]$$

From Equation (9), if the intensity of received light beam is modulated by a single frequency signal, the amplitude values of the certain spectrum lines output from the four APDs will also reflect the energy distribution ratio of the beacon light. Therefore, the line spectrum detection method can be used to extract the amplitudes of the photocurrents, and the amplitudes can be substituted into Equation (1) to calculate the spot position.

After intensity modulating, the spectrum of the photocurrent signal output by QD is a line spectrum signal. In contrast, the energy of Gaussian noise is distributed over a wider frequency band. In addition, the amplitude value of the spectrum line at this frequency point is proportional to the energy of the beam incident on the QD target surface. Therefore, the amplitude of the spectrum line at the frequency point Ω can be detected by fast Fourier transform (FFT) and substituted into Equation (1) to calculate the spot position.

However, the modulation only concentrates the valid signal energy and improves the SNR at the local frequency point, but cannot suppress noise. Under the condition of extremely low SNR, the interference of strong noise will greatly reduce the detection accuracy of the direct FFT method. Therefore, this paper proposes to use the TRC algorithm with window function to suppress noise, and then perform the FFT operation, which can provide a higher SNR gain and achieve the purpose of accurately detecting the spectrum line amplitude of the weak signal.

## 4. TRC Based Photocurrent Amplitude Detection

### 4.1. Time Reversal Convolution Algorithm

The time reversal convolution operation process can be expressed as [24]:

(10) $$\begin{array}{ll} F_{x}(t) & =x(-t)*x(t)\\ & =[s(-t)+n(-t)]*[s(t)+n(t)]\\ & =s(-t)*s(t)+s(-t)*n(t)+n(-t)*s(t)+n(-t)*n(t)\\ & =F_{ss}(t)+F_{sn}(t)+F_{ns}(t)+F_{nn}(t) \end{array}$$

If the signal *s*(*t*) and the noise *n*(*t*) are independent of each other, then *F_sn_* and *F_ns_* are approximately equal to 0, so Equation (10) is simplified as:

(11) $$F_{x}(t)\approx F_{ss}(t)+F_{nn}(t)$$

Define a signal *x*(*t*) with noise as:

(12) x(t)=s(t)+n(t)=Acos(2π⋅f⋅t+φ)+n(t)

where *A* is the signal amplitude, *f* is the signal frequency, *s*(*t*) is the signal phase, and *n*(*t*) is Gaussian noise with a mean of 0 and a variance of $\sigma_{n}^{2}$. Then, *F_ss_*(*t*) can be written as:

(13) $$F_{ss}(t)=\left\{ \begin{array}{l} \frac{A^{2}(T+t)}{2}\cos(2\pi \cdot f\cdot t)\;-T\leq t\leq 0\\ \frac{A^{2}(T-t)}{2}\cos(2\pi \cdot f\cdot t)\;0<t\leq T \end{array}\right.$$

If the power spectrum density of *n*(*t*) is *P_0_*, the TRC operation result of *n*(*t*) is:

(14) $$F_{nn}(t)=P_{0}\delta (t)$$

**Figure 2:**
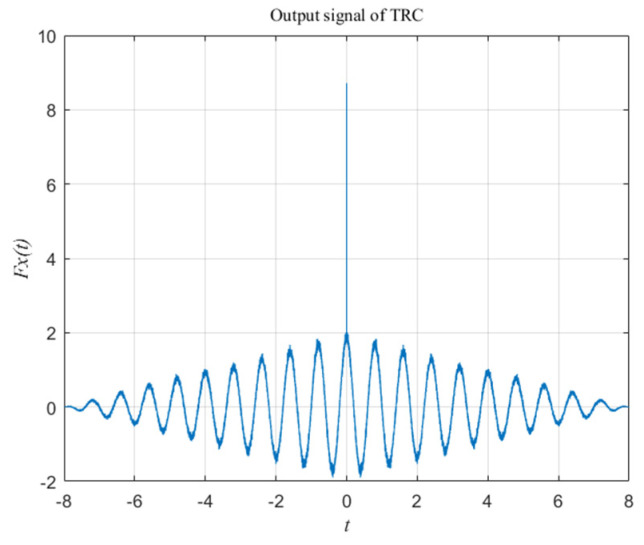
Result of the time reversal convolution (TRC) operation.

So, the result of TRC operation of *x*(*t*) can be shown as the curve in Figure 2.

**Figure 3:**
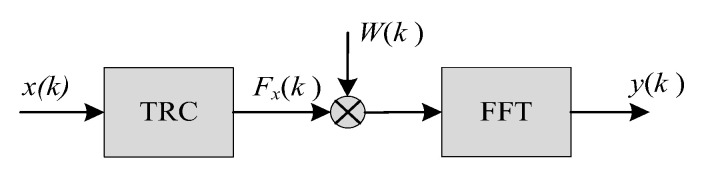
Line spectrum detection based on TRC algorithm.

Figure 2 shows that after TRC operation, the energy distribution of *F_ss_*(*t*) and *F_nn_*(*t*) are different. The energy of *s*(*t*) is still contained in the cosine signal of the same frequency, distributed throughout the whole convolution time. The energy of *n*(*t*) is concentrated at the convolution time point *t* = 0. If *F_x_*(0) is set to 0, most of the noise energy can be removed at the cost of less valid signal energy. After using the TRC method to suppress the noise, the output signal is transformed by the fast Fourier transform (FFT) operation, which can help us to focus on detecting the amplitude at the certain frequency point, thereby avoiding the interference of harmonics. The processing flow of the proposed method is shown in Figure 3.

In Figure 3, *W*(*k*) is the weighted denoising window function, which is written as:

(15) $$W(k)=\left\{ \begin{array}{l} 1\;k\neq 0\\ 0\;k=0 \end{array}\right.$$

### 4.2. SNR Gain of TRC Algorithm

Discretize the continuous signal *x*(*t*) and rewrite *x*(*t*) as:

(16) $$x(k)=s(k)+n(k)=A\cos(2\pi \cdot f\cdot kT_{s}+\varphi )+n(k)\;k=0,\;1,\;2,\cdots ,N-1$$

Then, the SNR of *x*(*k*) is:

(17) $$SNR_{x}=\frac{P_{s}}{P_{n}}=\frac{A^{2}}{2\sigma_{n}^{2}}$$

The expression of *F_ss_*(*k*) is

(18) $$F_{ss}(k)=\left\{ \begin{array}{l} \frac{A^{2}(N+k)}{2}\cos(2\pi \cdot f\cdot kT_{s})\;k=-N+1,\cdots ,\;0\\ \frac{A^{2}(N-k)}{2}\cos(2\pi \cdot f\cdot kT_{s})\;k=1,\;2,\cdots ,\;N-1 \end{array}\right.$$

To suppress noise, we set *F_x_*(0) to 0. Thus the noise and the effective signal both lose part of the energy, so the signal power *F_x_*(*k*) component power $P_{Fs}$ and noise power $P_{Fn}$ are calculated as:

(19) $$P_{Fs}=\frac{1}{2N-1}\left(\sum_{k=-N+1}^{-1}{\left|F_{ss}(k)\right|}^{2}+\sum_{k=1}^{N-1}{\left|F_{ss}(k)\right|}^{2}\right)=\frac{A^{4}N\cdot (N-3)}{24}$$

(20) $$P_{Fn}=\frac{1}{2N-1}\left(\sum_{k=-N+1}^{-1}{\left|F_{nn}(k)\right|}^{2}+\sum_{k=1}^{N-1}{\left|F_{nn}(k)\right|}^{2}\right)=\frac{\sigma_{n}^{4}N}{2}$$

So, the output SNR of TRC operation is:

(21) $$SNR_{F}=\frac{N-3}{3}SNR_{x}^{2}$$

Then, the SNR gain is:

(22) $$G_{F}=\frac{SNR_{F}}{SNR_{x}}=\frac{N-3}{3}SNR_{x}$$

### 4.3. Simulation

Define a cosine signal with noise as

(23) $$V(t)=a_{1}\cdot \cos(2\pi \cdot f_{1}t+\varphi_{1})+n_{1}(t)$$

The main parameters are: amplitude *a*_1_ = 0.001; frequency *f*_1_ = 125 kHz; sampling frequency *f_s_* = 100 MHz; *n*_1_(t) is the Gaussian white noise with a mean of 0. The data length *N* involved in the calculation is 8000 points.

**Figure 4:**
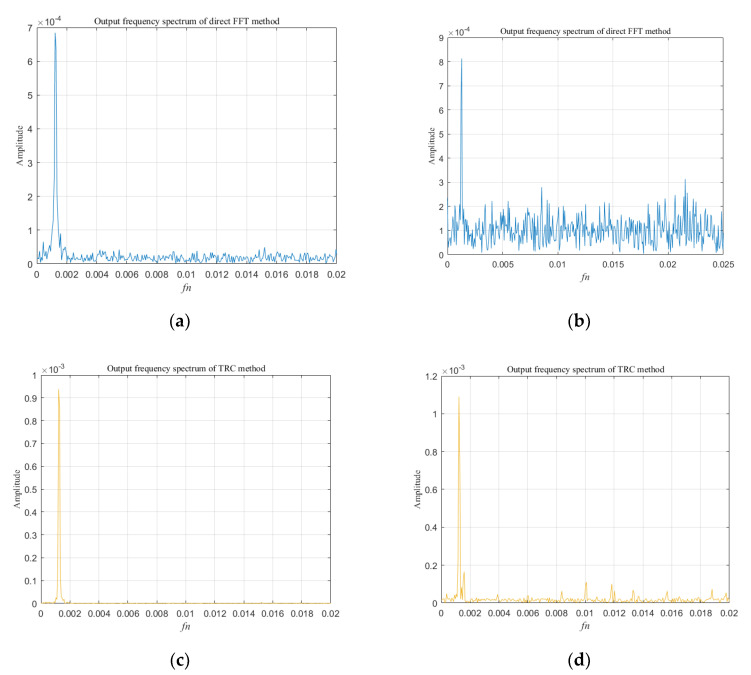
The frequency spectrum of the output signal of direct fast Fourier transform (FFT) method and TRC method under different signal-to-noise ratio (SNR) conditions. (**a**) When SNR = −5 dB, direct FFT method output spectrum; (**b**) When SNR = −20 dB, direct FFT method output spectrum; (**c**) When SNR = −5 dB, TRC method output spectrum; (**d**) When SNR = −20 dB, TRC method output spectrum.

Under different SNR conditions, we used the TRC method and the direct FFT method to detect the line spectrum amplitude of *V*(*t*), and the results are shown in Figure 4. When SNR = −5 dB, the spectrum obtained by the TRC method (as showed Figure 4c) and the direct FFT method (as showed Figure 4a) both highlight the spectrum line well at the frequency of 125 kHz. However, when SNR = −20 dB, the amplitude of the spectrum line at 125 kHz obtained by the direct FFT method drops sharply (as showed Figure 4b). In contrast, the TRC method can effectively suppress noise, so that the spectrum line of *V*(*t*) is highlighted in the strong noise. As Figure 4d shows, the spectrum line of *V*(*t*) has been significantly enhanced at 125 kHz, and the output SNR of the TRC algorithm has been increased to 2.25 dB.

It can be seen from Figure 4 that the noise floor in the frequency spectrum of the direct FFT method is much larger than that of the TRC method, and as the SNR decreases, the difference in the noise floor becomes larger. Therefore, the amplitude estimated by the two methods is quite different. In Figure 4, the abscissa *f_n_* is the normalized frequency: *f_n_*= *f*/*f_s_*, where *f* is the signal frequency and *f_s_* is the sampling frequency.

**Figure 5:**
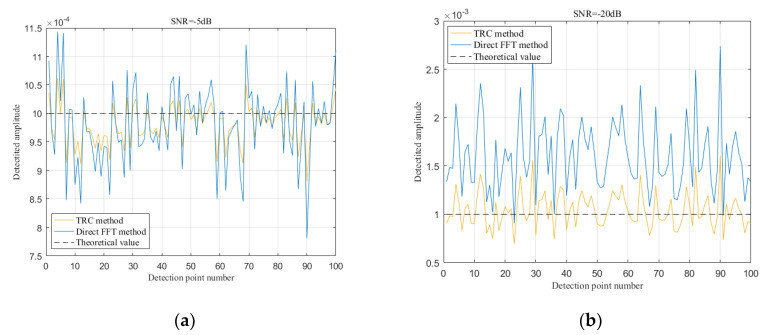
The amplitude detected by direct FFT method and TRC method under different SNR conditions. (**a**) When SNR = −5 dB, detected amplitude values of *V*(*t*); (**b**) When SNR = −20 dB, detected amplitude values of *V*(*t*).

In Figure 5, the abscissa is the detection point number. This figure shows the amplitude values detected by the two methods under different SNR conditions. When SNR = −5 dB, the average relative error of the direct FFT method is 28.1%, and the average relative error of the TRC method is 15.9%. When SNR = −20 dB, the average relative error of the direct FFT method reaches 60.9%, while the average relative error of the TRC method is limited within 16.9%. Therefore, the TRC method has better detection accuracy than the direct FFT method.

The average relative error is defined as

(24) $$\delta_{AVG}=\frac{1}{N}\sum_{i=1}^{N}\left(\left|x_{0}-X\right|/X\right)\cdot 100\%$$

where *x*_0_ is the detected value, *X* is the theory value.

**Figure 6:**
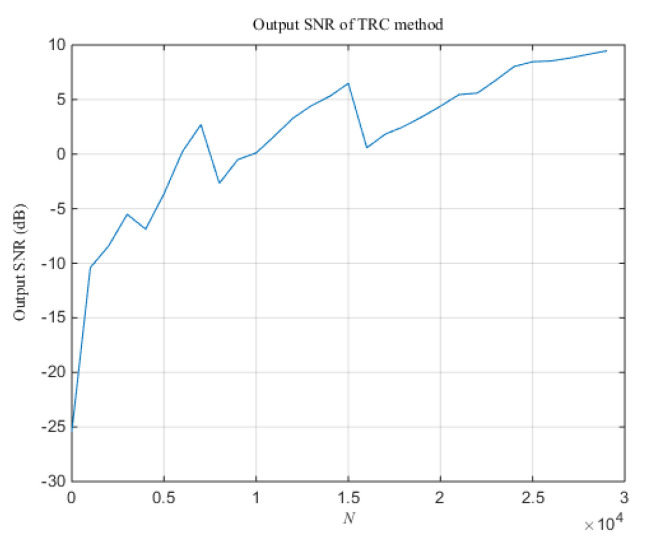
The change curve of the output SNR of the TRC method following *N*.

In addition, Figure 6 shows the change curve of the output SNR of the TRC method following the data length *N* when the SNR is −20 dB. It can be seen that the TRC algorithm with window function can effectively suppress noise and improve the SNR of the output signal. As *N* gradually increases, the output SNR of the TRC method also increases, which can improve the accuracy of spot position detection. However, an excessively large amount of data will increase the computational burden, which will weaken the real-time performance of spot position detection. Therefore, the appropriate data length must be selected according to the accuracy and real-time requirements of the system. Considering factors such as algorithm calculation efficiency and SNR gain, the value of data length *N* is chosen to be 8000 in the subsequent experiments.

## 5. Experiment

**Figure 7:**
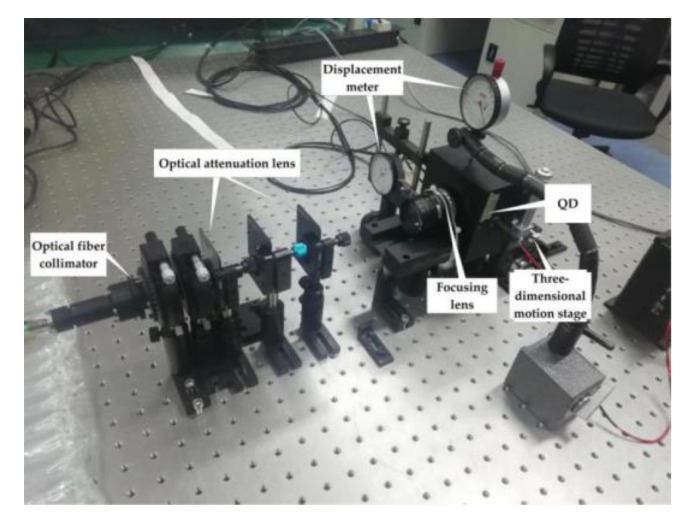
The experiment platform.

The experimental optical platform is shown in Figure 7. The platform used a tunable semiconductor laser with a wavelength of 1550 nm and an output power of 120 μW. The frequency of the modulation signal is 125 kHz. The laser is emitted from the collimator, passes through the attenuation glass, enters the lens, and finally illuminates the QD target surface. The power of the incident light beam is 1.39~13.73 nW. The QD with target radius of 1 mm and a gap width of 0.01 mm is placed on a three-dimensional stage, so that the position of the spot on the QD target surface can be adjusted. Furthermore, QD can be moved back and forth to adjust the size of the incident spot. Two displacement meters are placed on each side of the translation stage, which can measure the displacement of the QD in the x-axis direction and the y-axis direction.

The ADC model used in the platform is AD9653 whose sampling frequency is 100 MHz and output data width is 16 bits. The data length used in the experiment is 8000 points. Utilizing the high-speed sampling capability of ADC and the parallel computing capability of FPGA can better meet the real-time requirements of the ATP system and avoid the interference caused by spacecraft dynamics and jitter.

**Figure 8:**
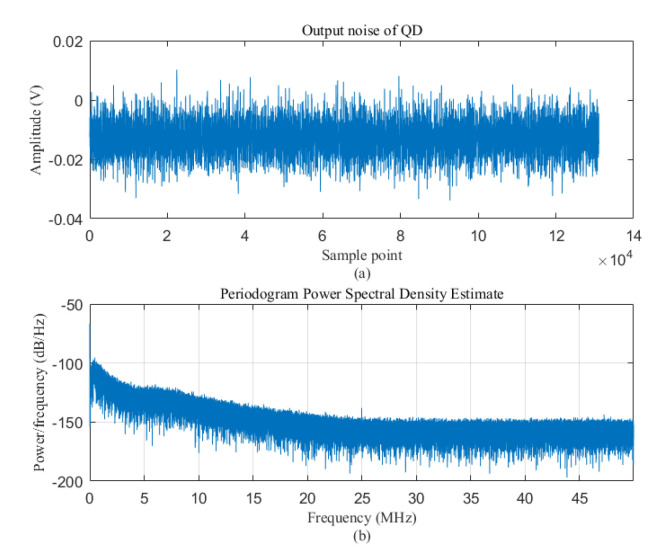
The output noise of the QD and its power spectral density (PSD).

Figure 8 shows the pure noise signal and its power spectral density output by the detector used in the experiment under the condition of no incident light irradiation. It can be seen from the figure that, after removing the DC component, the power spectral density of the noise is approximately evenly distributed over the entire frequency band.

**Figure 9:**
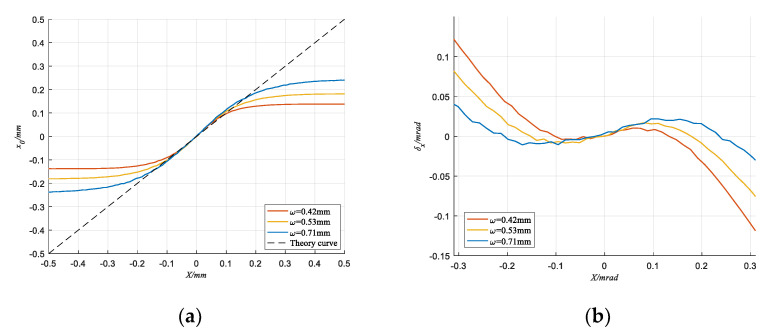
When SNR = −7.66 dB, detection results of TRC method under different spot radius. (**a**) The Calculated coordinate curve; (**b**) Absolute error.

Figure 9 shows the coordinate curve and error curve obtained by TRC method under different spot radius when QD output SNR = −7.66 dB. In Figure 9a, ω is the spot radius, the abscissa *X* is the real coordinate value of the light spot, and the ordinate *x_0_* is the calculated coordinate value. In Figure 9b, *δ_x_* is the absolute error of the calculated coordinate. Taking x coordinates as an example, the absolute error is denoted as:

(25) $$\delta_{x}=\frac{x_{0}-X}{D}$$

where *D* is the focal length of the system.

It can be seen that all three coordinate curves change in an “S” shape, which is affected by spot radius, QD’s target size and gap width. However, the detection curve of the TRC method still maintain good linearity and the absolute errors are all limited in 0.0176 mrad in the coordinate interval (−0.2 mrad, 0.2 mrad). This means that the performance of the TRC algorithm has high stability under different spot radius conditions, so the detection results of the TRC method can be relatively easily corrected for the non-linearity by interpolating, fitting and other methods. In order to avoid the influence of QD’s non-linearity, this paper selects the data in the (−0.1 mm, 0.1 mm) coordinate interval to calculate various detection errors.

**Figure 10:**
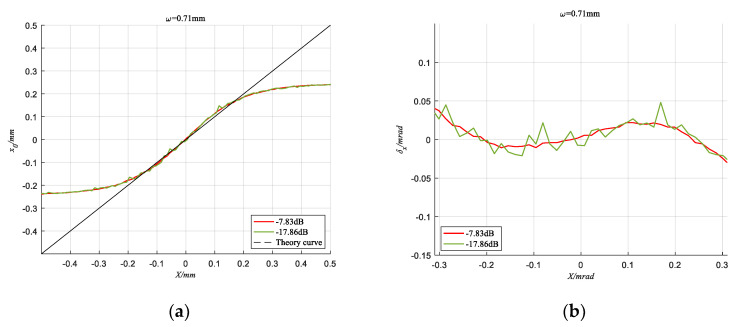
When *ω* = 0.71 mm, detection results of TRC method under different SNR conditions. (**a**) The Calculated coordinate curve; (**b**) Absolute error.

**Figure 11:**
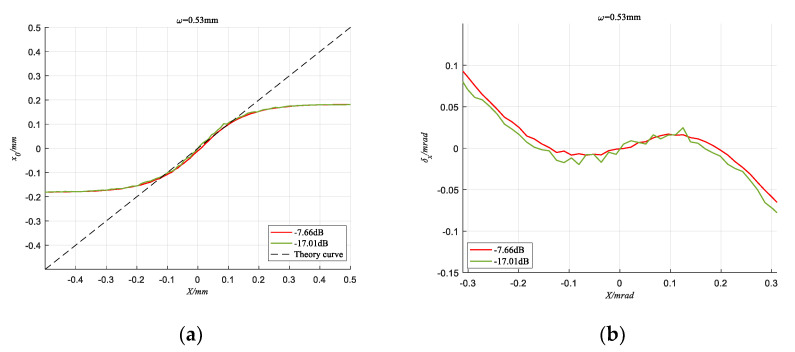
When *ω* = 0.53 mm, detection results of TRC method under different SNR conditions. (**a**) The Calculated coordinate curve; (**b**) Absolute error.

When the spot radius ω = 0.53 mm and ω = 0.71 mm respectively, Figure 10 and Figure 11 show the detection curves under different SNR conditions. It can be seen that the detection result of the TRC method can also present a smoother curve when the SNR is as low as −17.86 dB, and the absolute error fluctuation is suppressed within 0.0238 mrad. This shows that the TRC method can effectively suppress noise and improve the detection accuracy of the spot position.

**Figure 12:**
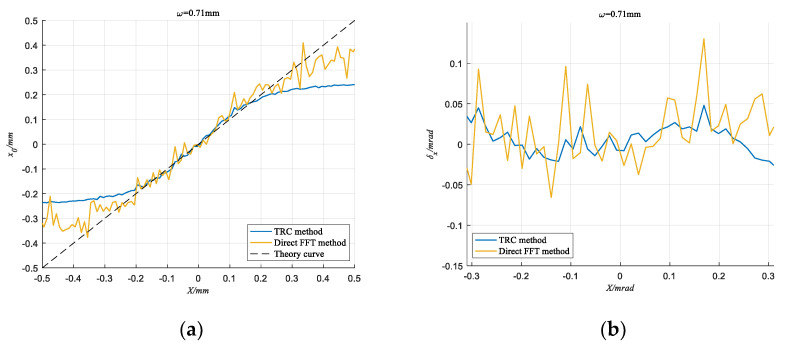
When *ω* = 0.71 mm and SNR = −17.86 dB, detection results of TRC method and direct FFT method. (**a**) The Calculated coordinate curve; (**b**) Absolute error.

**Figure 13:**
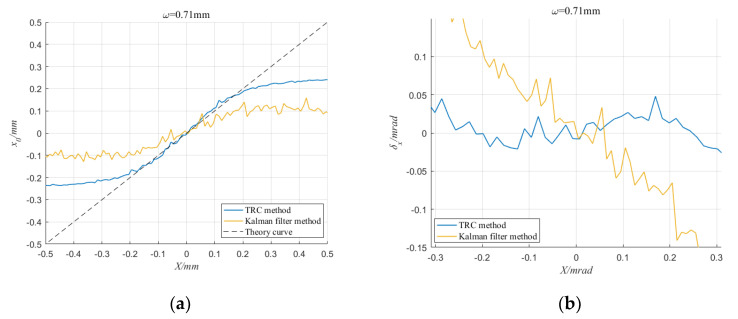
When ω = 0.71 mm and SNR= −17.86 dB, detection results of TRC method and Kalman filter method. (**a**) The Calculated coordinate curve; (**b**) Absolute error.

In contrast, under the same conditions, we also used the direct FFT method and the Kalman filter method to detect the spot position. Figure 12 and Figure 13 show the output results of the direct FFT method, the Kalman filter method and the TRC method when the SNR is −17.86 dB and the spot radius is 0.71 mm. It can be seen that the detection curve of the TRC method is significantly smoother than that of the direct FFT method, and the Kalman filter method cannot detect the spot position correctly at all. This means that the TRC method has a strong ability to suppress random noise. 

In addition, we use maximum error and root-mean-square error to compare the performance of the two methods. Maximum error is defined as:

(26) $$\delta_{\max}=\underset{i}{\max}(|\delta_{xi}|)$$

and root-mean-square error is defined as:

(27) $$\delta_{RMSE}=\sqrt{\sum_{i=1}^{N}\delta_{xi}^{2}/N}$$

The comparison between the two methods is shown in Table 1. When the SNR is relatively high, the two methods have little difference in the ability to suppress noise, and the direct FFT method is slightly worse. However, when the QD output SNR is only −17.86 dB, the errors of the direct FFT method increase sharply. In contrast the root-mean-square error of the TRC method is still less than 0.0135 mrad, and the maximum error is also limited within 0.0238 mrad.

## 6. Discussion

This article mainly discusses the problem of improving QD’s detection accuracy of spot position when the SNR is lower than −17 dB. The key to solving this problem is to provide a sufficiently large SNR gain, so the paper considers using the TRC line spectrum enhancement algorithm to highlight the spectrum of the photocurrent signal of QD. This proposed method changes the energy distribution of the effective signal and broadband noise in the time-domain, and uses a window function to suppress most of the noise, which is very different from the traditional filtering method. It can be seen from Equation (16) that when the length of the sequence *x*(*k*) tends to infinity, the noise energy is all concentrated at time 0, and the window function has the greatest suppression of noise. Therefore, the SNR gain provided by the TRC algorithm is proportional to the length of the sequence *x*(*k*). However, if the length *N* of the sequence *x*(*k*) is unreasonably selected, resulting in the exact opposite phase between *x*(*k*) and *x*(-*k*), the time-domain correlation between the two sequences will be reduced, thus the value of each element in *F_x_*(*k*) will decrease. This will cause the output SNR of the TRC algorithm to suddenly drop (as shown in Figure 6). Therefore, when choosing the sequence length *N*, must consider the sequence *x*(*k*) contains an integer number of the valid signal cycle.

Moreover, it can be seen from Figure 2 that at time 0, most of the noise energy is concentrated, but the energy of the valid signal is also the largest here. Therefore, the window function suppresses noise while also losing greater valid signal energy. This is equivalent to indirect loss of beam energy, resulting in a decrease in the effective radius of the Gaussian spot, and aggravating the nonlinearity of the detection curve, and reducing the detection range of the QD. The linearity can be improved by fitting or interpolation, and a more suitable window function should be found to reduce the loss of effective signal.

## 7. Conclusions

In inter-satellite optical communication terminals without independent beacon light, the energy of the beacon light allocated by the ATP system is very small, which makes the output SNR of QD often lower than −17 dB. Thus, the QD-based spot position detection accuracy seriously degrades. Therefore, we propose the TRC algorithm with window function to suppress strong noise and enhance the line spectrum of the weak photocurrent signal to accurately detect the photocurrent amplitude.

This method uses a cosine signal to modulate the intensity of the light beam, so that each photocurrent signal output by the QD becomes a line spectrum signal. Then, the TRC operation concentrates the energy of the broadband noise to the time 0 point, and the energy of the valid signal is distributed to the entire envelope of the output result. Therefore, the noise can be effectively removed by the window function, and then the FFT operation is used to obtain the amplitude value of the line spectrum signal. The TRC method with window function can use a small amount of data to obtain a high SNR gain, thereby improving the spot position detection accuracy under extremely low SNR conditions. In the experiment, when the output SNR of the QD is as low as −17.86 dB, the proposed method can still detect the spot position, and the max error is limited within 0.0238 mrad. Through simulation and experimental comparison, the detection accuracy of the proposed method under the condition of extremely low SNR is obviously better than that of direct FFT method and Kalman filter method.

The SNR gain of the TRC method depends on the data length used in the calculation, but the real-time detection of the system must also be considered in practical applications. Therefore, considering the two factors of the SNR gain and the real-time performance, the data length used in the experiment is 8000 points, and relatively good results have been obtained. Furthermore, the TRC algorithm does not need to consider signal synchronization. These advantages are very important in inter-satellite laser communication that requires high real-time performance.

## Figures and Tables

**Table 1:** The errors in different spot radius and SNR conditions.

Spot Radius (mm)	SNR (dB)	TRC Method		Direct FFT Method	
		δ_max_ (mrad)	δ_RMSE_ (mrad)	δ_max_ (mrad)	δ_RMSE_ (mrad)
0.42	−5.93	0.0124	0.0065	0.0131	0.0063
	−14.58	0.0138	0.0069	0.0443	0.0212
0.53	−7.66	0.0132	0.0076	0.0141	0.0078
	−17.01	0.0181	0.0112	0.0576	0.0272
0.71	−7.83	0.0176	0.0109	0.0243	0.0135
	−17.86	0.0238	0.0135	0.0960	0.0378
